# Supplementary material for: Food insecurity and psychological stress among migrants and refugees in high-income countries: Protocol for a systematic review and meta-analysis
Source: PLoS One. 2024 Dec 5;19(12):e0311796. doi: 10.1371/journal.pone.0311796 (PMC11620644; doi:10.1371/journal.pone.0311796)
Supplement: S2 File — (DOCX) [file pone.0311796.s002.docx]

**Supplement FILE 2**

**Table 2. MEDLINE Search Strategy (Via OVID)**

| **Database** | **Search terms** | **Items found** |
| --- | --- | --- |
| **Food insecurity and mental stress** | | |
| **#1** | "Transients and Migrants"/ | **14578** |
| **#2** | exp "Emigrants and Immigrants"/ | **15947** |
| **#3** | Refugees/ | **13570** |
| **#4** | (migrant* or immigrant* or emigrant* or asylum seeker* or refugee* or displaced person* or undocumented migrant*).mp. | **77755** |
| **#5** | 1 or 2 or 3 or 4 | **77755** |
| **Psychological Stress** | | |
| **#6** | stress, psychological/ or burnout, psychological/ or financial stress/ | **137930** |
| **#7** | Anxiety/ | **110614** |
| **#8** | Depression/ | **155146** |
| **#9** | Mental Health/ | **65284** |
| **#10** | Psychological Distress/ | **4423** |
| **#11** | Financial Stress/ | **1192** |
| **#12** | stress, psychological/ or burnout, psychological/ or financial stress/ | **137930** |
| **#13** | (psychological stress* or psychological distress* or burn out* or anxiety* or depression* or mental health* or financial stress* or psychological burn out*).mp. | **927896** |
| **#14** | 6 or 7 or 8 or 9 or 10 or 11 or 12 or 13 | **1010896** |
| **Food Insecurity** | | |
| **#15** | exp food insecurity/ or access to healthy foods/ | **1620** |
| **#16** | exp food security/ or access to healthy foods/ | **770** |
| **#17** | Hunger/ | **6114** |
| **#18** | Food Deprivation/ | **8739** |
| **#19** | (Food insecurity* or hunger* or food deprivation* or food poverty* or food scarcity* or inadequate food access* or food security* or food crisis*).mp. | **44733** |
| **#20** | 15 or 16 or 17 or 18 or 19 | **44752** |
| **High-Income Countries** | | |
| **#21** | (America* or Andorra* or Antigua* or Aruba* or Australia* or Austria* or Barbuda* or Bermuda* or Britain or British or Baham* or Bahrain* or Barbad* or Belgium or Belgian* or Brunei* or Canada or Canadian* or Cayman Island* or Channel Island* or Chile* or Croatia* or Curacao* or Cyprus or Cyprian* or Cypriot? or Czech* or Darussalam or Denmark or Danish or England or English or Estonia* or Faroe Island* or Finland or Finnish or Finn? or France or French or German* or Gibralta* or Greece or Greek* or Greenland* or Guam* or Hong Kong* or Hungary or Hungarian* or Iceland* or Ireland or Irish or "Isle of Man" or Israel* or Italy or Italian* or Japan* or South Korea* or Kuwait* or Latvia* or Liechtenstein* or Lithuania* or Luxembourg* or Macao* or Malta or Maltese or Monaco or Nauru* or Netherlands or Dutch or New Caledonia* or New Zealand* or Northern Mariana Island* or Norway or Norwegian* or Oman* or Panama* or Poland or Polish or Portug* or Puerto Ric* or Romania* or Qatar* or Saint Kitts or San Marino or Saint Martin or Sint Maarten or Saudi Arabia* or Seychelles or Singapore* or Slovak* or Slovenia* or Spain or Spanish or Sweden or Swedish or Switzerland or Swiss or Taiwan* or Trinidad* or Tobago* or (Turks and Caicos Island*) or United Arab Emirates or United Kingdom or UK or United States or USA or Uruguay* or Virgin Island* or (western adj (country* or economy* or nation*))).mp. | **5958671** |
| **#22** | (America* or Andorra* or Antigua* or Aruba* or Australia* or Austria* or Barbuda* or Bermuda* or Britain or British or Baham* or Bahrain* or Barbad* or Belgium or Belgian* or Brunei* or Canada or Canadian* or Cayman Island* or Channel Island* or Chile* or Croatia* or Curacao* or Cyprus or Cyprian* or Cypriot? or Czech* or Darussalam or Denmark or Danish or England or English or Estonia* or Faroe Island* or Finland or Finnish or Finn? or France or French or German* or Gibralta* or Greece or Greek* or Greenland* or Guam* or Hong Kong* or Hungary or Hungarian* or Iceland* or Ireland or Irish or "Isle of Man" or Israel* or Italy or Italian* or Japan* or South Korea* or Kuwait* or Latvia* or Liechtenstein* or Lithuania* or Luxembourg* or Macao* or Malta or Maltese or Monaco or Nauru* or Netherlands or Dutch or New Caledonia* or New Zealand* or Northern Mariana Island* or Norway or Norwegian* or Oman* or Panama* or Poland or Polish or Portug* or Puerto Ric* or Romania* or Qatar* or Saint Kitts or San Marino or Saint Martin or Sint Maarten or Saudi Arabia* or Seychelles or Singapore* or Slovak* or Slovenia* or Spain or Spanish or Sweden or Swedish or Switzerland or Swiss or Taiwan* or Trinidad* or Tobago* or (Turks and Caicos Island*) or United Arab Emirates or United Kingdom or UK or United States or USA or Uruguay* or Virgin Island* or (western adj (countr* or econom* or nation*))).sh. | **2831148** |
| **#23** | ((high* or upper) adj5 income? adj5 (countr* or econom* or group? or nation?)).mp. | **19856** |
| **#24** | 21 or 22 or 23 | **5971068** |
| **#25** | 5 and 14 and 20 and 24 | **64** |
|  |  |  |

**MEDLINE Search Strategy (Via OVID)**

| **Database** | **Search terms** | **Items found** |
| --- | --- | --- |
| **Food insecurity& Mental stress** | | |
| **#1** | "Transients and Migrants"/ | **14578** |
| **#2** | exp "Emigrants and Immigrants"/ | **15947** |
| **#3** | Refugees/ | **13570** |
| **#4** | (migrant* or immigrant* or emigrant* or asylum seeker* or refugee* or displaced person* or undocumented migrant*).mp. | **77755** |
| **#5** | 1 or 2 or 3 or 4 | **77755** |
| **Another search** | | |
| **#6** | stress, psychological/ or burnout, psychological/ or financial stress/ | **137930** |
| **#7** | Anxiety/ | **110614** |
| **#8** | Depression/ | **155146** |
| **#9** | Mental Health/ | **65284** |
| **#10** | Psychological Distress/ | **4423** |
| **#11** | Financial Stress/ | **1192** |
| **#12** | stress, psychological/ or burnout, psychological/ or financial stress/ | **137930** |
| **#13** | (psychological stress* or psychological distress* or burn out* or anxiety* or depression* or mental health* or financial stress* or psychological burn out*).mp. | **927896** |
| **#14** | 6 or 7 or 8 or 9 or 10 or 11 or 12 or 13 | **1010896** |
| **ANOTHER SEARCH** | | |
| **#15** | exp food insecurity/ or access to healthy foods/ | **1620** |
| **#16** | exp food security/ or access to healthy foods/ | **770** |
| **#17** | Hunger/ | **6114** |
| **#18** | Food Deprivation/ | **8739** |
| **#19** | (Food insecurity* or hunger* or food deprivation* or food poverty* or food scarcity* or inadequate food access* or food security* or food crisis*).mp. | **44733** |
| **#20** | 15 or 16 or 17 or 18 or 19 | **44752** |
| **Another Search** | | |
| **#21** | (America* or Andorra* or Antigua* or Aruba* or Australia* or Austria* or Barbuda* or Bermuda* or Britain or British or Baham* or Bahrain* or Barbad* or Belgium or Belgian* or Brunei* or Canada or Canadian* or Cayman Island* or Channel Island* or Chile* or Croatia* or Curacao* or Cyprus or Cyprian* or Cypriot? or Czech* or Darussalam or Denmark or Danish or England or English or Estonia* or Faroe Island* or Finland or Finnish or Finn? or France or French or German* or Gibralta* or Greece or Greek* or Greenland* or Guam* or Hong Kong* or Hungary or Hungarian* or Iceland* or Ireland or Irish or "Isle of Man" or Israel* or Italy or Italian* or Japan* or South Korea* or Kuwait* or Latvia* or Liechtenstein* or Lithuania* or Luxembourg* or Macao* or Malta or Maltese or Monaco or Nauru* or Netherlands or Dutch or New Caledonia* or New Zealand* or Northern Mariana Island* or Norway or Norwegian* or Oman* or Panama* or Poland or Polish or Portug* or Puerto Ric* or Romania* or Qatar* or Saint Kitts or San Marino or Saint Martin or Sint Maarten or Saudi Arabia* or Seychelles or Singapore* or Slovak* or Slovenia* or Spain or Spanish or Sweden or Swedish or Switzerland or Swiss or Taiwan* or Trinidad* or Tobago* or (Turks and Caicos Island*) or United Arab Emirates or United Kingdom or UK or United States or USA or Uruguay* or Virgin Island* or (western adj (countr* or econom* or nation*))).mp. | **5958671** |
| **#22** | (America* or Andorra* or Antigua* or Aruba* or Australia* or Austria* or Barbuda* or Bermuda* or Britain or British or Baham* or Bahrain* or Barbad* or Belgium or Belgian* or Brunei* or Canada or Canadian* or Cayman Island* or Channel Island* or Chile* or Croatia* or Curacao* or Cyprus or Cyprian* or Cypriot? or Czech* or Darussalam or Denmark or Danish or England or English or Estonia* or Faroe Island* or Finland or Finnish or Finn? or France or French or German* or Gibralta* or Greece or Greek* or Greenland* or Guam* or Hong Kong* or Hungary or Hungarian* or Iceland* or Ireland or Irish or "Isle of Man" or Israel* or Italy or Italian* or Japan* or South Korea* or Kuwait* or Latvia* or Liechtenstein* or Lithuania* or Luxembourg* or Macao* or Malta or Maltese or Monaco or Nauru* or Netherlands or Dutch or New Caledonia* or New Zealand* or Northern Mariana Island* or Norway or Norwegian* or Oman* or Panama* or Poland or Polish or Portug* or Puerto Ric* or Romania* or Qatar* or Saint Kitts or San Marino or Saint Martin or Sint Maarten or Saudi Arabia* or Seychelles or Singapore* or Slovak* or Slovenia* or Spain or Spanish or Sweden or Swedish or Switzerland or Swiss or Taiwan* or Trinidad* or Tobago* or (Turks and Caicos Island*) or United Arab Emirates or United Kingdom or UK or United States or USA or Uruguay* or Virgin Island* or (western adj (countr* or econom* or nation*))).sh. | **2831148** |
| **#23** | ((high* or upper) adj5 income? adj5 (countr* or econom* or group? or nation?)).mp. | **19856** |
| **#24** | 21 or 22 or 23 | **5971068** |
| **#25** | 5 and 14 and 20 and 24 | **64** |
|  |  |  |
